# The role and novel use of natural killer cells in graft-versus-leukemia reactions after allogeneic transplantation

**DOI:** 10.3389/fimmu.2024.1358668

**Published:** 2024-05-16

**Authors:** Ashley D. Hadjis, Shannon R. McCurdy

**Affiliations:** ^1^ Department of Internal Medicine, Hospital of the University of Pennsylvania, Philadelphia, PA, United States; ^2^ Abramson Cancer Center and the Division of Hematology and Oncology, Hospital of the University of Pennsylvania, Philadelphia, PA, United States

**Keywords:** NK cell, graft-versus-leukemia, allogeneic transplant, PTCy, alloreactivity, KIR

## Abstract

Allogeneic hematopoietic cell transplantation (HCT) has transformed over the past several decades through enhanced supportive care, reduced intensity conditioning (RIC), improved human leukocyte antigen (HLA) typing, and novel graft-versus-host disease (GVHD)-prevention and treatment strategies. Most notably, the implementation of post-transplantation cyclophosphamide (PTCy) has dramatically increased the safety and availability of this life-saving therapy. Given reductions in nonrelapse mortality (NRM) with these advances, the HCT community has placed even greater emphasis on developing ways to reduce relapse - the leading cause of death after HCT. When using RIC HCT, protection from relapse relies predominantly on graft-versus-leukemia (GVL) reactions. Donor lymphocyte infusion (DLI), adoptive cellular therapy, checkpoint inhibition, and post-HCT maintenance strategies represent approaches under study that aim to augment or synergize with the GVL effects of HCT. Optimizing donor selection algorithms to leverage GVL represents another active area of research. Many of these strategies seek to harness the effects of T cells, which for decades were felt to be the primary mediators of GVL and the focus of investigation in relapse reduction. However, there is growing interest in capitalizing on the ability of natural killer (NK) cells to yield potent anti-tumor effects. A potential advantage of NK cell-based approaches over T cell-mediated is the potential to reduce NRM in addition to relapse. By decreasing infection, without increasing the risk of GVHD, NK cells may mitigate NRM, while still yielding relapse reduction through identification and clearance of cancer cells. Most T cell-focused relapse-prevention strategies must weigh the benefits of relapse reduction against the increased risk of NRM from GVHD. In contrast, NK cells have the potential to reduce both, potentially tipping the scales significantly in favor of survival. Here, we will review the role of NK cells in GVL, optimization of NK cell match or mismatch, and burgeoning areas of research in NK cell therapy such as adoptive transfer and chimeric antigen receptor (CAR) NK cells.

## Introduction

Improved conditioning regimens and enhanced HLA typing have made allogeneic hematopoietic cell transplantation (HCT) safer. Whatsmore, the advent of modern GVHD prophylactic regimens has made HCT accessible to nearly every patient in need. In particular, PTCy has expanded the donor pool to safely include HLA-mismatched related (haplo) (1) and HLA-mismatched unrelated (MMUD) donors (2, 3). With reductions in nonrelapse mortality (NRM) through GVHD prevention, relapse remains the leading cause of HCT failure and the major unmet need in HCT (4). Unfortunately, therapeutic options to treat post-HCT relapse are limited and outcomes are generally poor (5). For instance, relapse after HCT accounts for 30% to 40% of deaths (6) and 1-year overall survival (OS) after acute myeloid leukemia (AML) relapse is only 23% (7). In HCT with PTCy, 1-year OS after relapse is between 37% to 46% (5). Typically, remission-inducing chemotherapy followed by DLI or second HCT has been associated with the best OS after post-HCT relapse and yet only 8-25% of patients achieve long-term survival with these therapies (8–10). Given these poor outcomes, it is important to better understand the biology of post-HCT relapse in order to both prevent and treat it.

There are multiple factors that have been associated with higher risk of post-HCT relapse, including lack of complete hematologic remission (i.e. CR) or presence of minimal residual disease (MRD) at time of HCT, high-risk cytogenetic or molecular characteristics, use of T cell-depleted (TCD) HCT platforms, and reduced-intensity conditioning (7, 11, 12). Currently, interventions to prevent post-HCT relapse span the pre-HCT, peri-HCT, and post-HCT settings. In the pre-HCT setting, efforts include novel therapies to achieve deeper remissions prior to HCT, better identification of MRD, and improvements in HCT conditioning (11, 13, 14). In the peri-HCT setting, strategies include optimization of graft cell dose, use of sirolimus rather than tacrolimus in post-HCT immunosuppression, and early and/or rapid immunosuppression taper (15–17). Finally, in the post-HCT setting, efforts are focused on pharmacologic or immunotherapeutic interventions such as maintenance chemotherapy or prophylactic DLI (8, 11, 14).

Acute myeloid leukemia (AML) is the most frequent indication for HCT because of the potency of the graft-versus-leukemia (GVL) effect mediated by donor-derived immune cells, chiefly natural killer (NK) and T cells, exerting anti-leukemic immune reactions (18). GVL was first described by Barnes et al. in 1956, when reporting in murine models cure of leukemia after total body irradiation (TBI) and HCT (19, 20). The clinical existence of GVL was then further demonstrated in a large registry study conducted by the International Bone Marrow Transplant Registry (IBMTR) (21). In that study, recipients of non-TCD allografts who developed acute GVHD (aGVHD), chronic GVHD (cGVHD), or both had decreased rates of relapse compared to recipients who never developed GVHD (21). A dose effect was demonstrated with increased severity of GVHD or presence of both acute and chronic GVHD being associated with the lowest relapse rates (21). These findings indicate that alloreactivity enhances GVL and illustrate the intimate relationship between GVHD and GVL, implicating cells involved in GVHD pathogenesis in mediation of GVL. In contrast to T cells, the main mediators of acute GVHD, NK cells also play a role in GVL, albeit without a significant association with GVHD development. Here, we review the immunobiology of GVL, with a focus on NK cells, and the current understanding of how NK cells might be harnessed to augment GVL to prevent post-HCT relapse.

## NK cell biology and role in the graft-versus-leukemia effect

NK cells are innate immune lymphocytes that identify and eliminate stressed cells (i.e. malignant or viral-infected cells) via direct lysis or production of proinflammatory cytokines. They account for 5 to 15% of peripheral blood lymphocytes and are the third most abundant lymphocyte lineage in the peripheral blood (22–26). NK cells can identify stressed cells through their expression of ligands associated with cellular stress (NKG2D or DNAM-1) or through absence of expression of HLA class 1 molecules (27, 28). NK cell activation is mediated by the balance of activating and inhibitory signals received upon contact with host or foreign cells: either via a lack of interaction between a NK cell inhibitory receptor and its cognate ligand, an interaction between a NK cell activating receptor and its cognate ligand, or a predominance of activating over inhibitory signals (27, 29, 30). The inhibitory receptors found on NK cells, which include inhibitory killer immunoglobulin-like receptors (iKIRs), CD94/NKG2A, and LILRB1 among many others (**Table 1**), interact with HLA class I molecules and promote self-tolerance, preventing the initiation of NK cell cytotoxicity (30). NK activation and cellular cytotoxicity occurs when activating receptors such as activating killer immunoglobulin-like receptors (aKIRs), NKp46, and NKG2D (**Table 1**), bind to their respective cognate ligands (27, 29). Human NK cells express KIRs, whereas murine NK cells express members of the Ly49 family of the C-type lectin type II transmembrane proteins, but these receptors are functionally analogous (31).

**Table 1:** Human NK Cell Activating and Inhibitory Receptors and their Ligands.

Receptor	Ligand
**Activating** NKG2D (CD314) NKG2C (CD159a) KIR2DL4 (CD158d) KIR2DS1 (CD158h) KIR2DS2 (CD158j) KIR2DS3 (CD158j) KIR2DS4 (CD158i) KIR2DS5 (CD158f) KIR3DS1 (CD158e1) NKp30 (CD337) NKp46 (CD335) NKp44 (CD336) DNAM-1 FcγRIII (CD16)	MIC-A/-B, ULBP1-4 HLA-E HLA-G HLA-C2 HLA-C1 Unknown HLA-A11, some HLA-C Unknown HLA-Bw4, HLA-F B7H6, BAT3/BAG6, pp65 of HCMV, PfEMP1 of Plasmodium falciparum, viral HA Heparin, viral HA and HN, CFP, PfEMP1 Viral VA and HN, PCNA, proteoglycans CD112, CD155 IgG
**Inhibitory** KIR2DL1 (CD158a) KIR2DL2 (CD158b) KIR2DL3 (CD158b) KIR2DL5 (CD158f) KIR3DL1 (CD158e1) KIR3DL2 (CD158k) NKR-P1A NKG2A/KLRD1 (CD159a/CD94) ILT2 (CD85) CD244(2B4) PD-1 (CD279) Siglec-7 (CD328) IRP60 (CD3000a) CD96 IL1R8 TIGIT TIM-3	HLA-C2 HLA-C1 HLA-C1 Unknown HLA-A-Bw4, HLA-B-Bw4 HLA-A3, HLA-A11 LLTI HLA-E HLA-A, HLA-B, HLA-C, HLA-G1, HCMV UL18 CD48 PDL-1 (CD274), PD-L2 (CD273) Ganglioside DSGb5 Alpha-herpes virus, Pseudorabide virus, Phosphatidylserine, Phosphatidylethanolamine PVR (CD155) IL-37 PVR (CD155) Gal-9, PtdSer, HMGB1, CEACAM1

**Figure 1:**
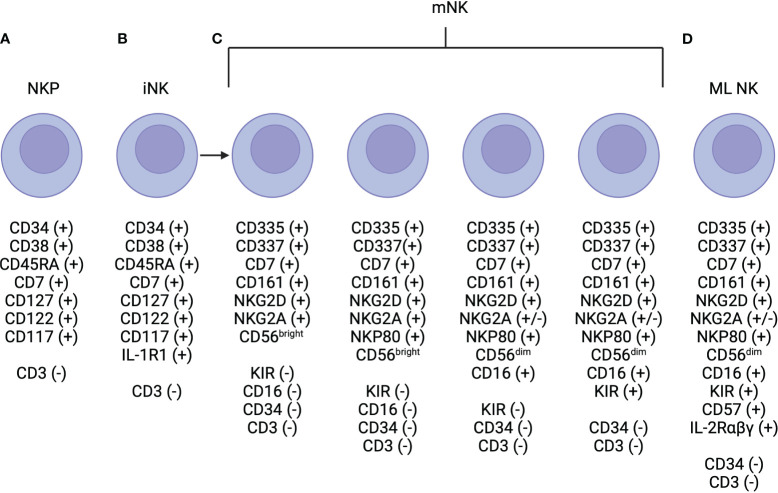
NK Cell Development. **(A)** Natural killer progenitor (NKP) cells are defined by are defined by expression of CD7, CD127, CD117 (c-Kit) and this marks the beginning of commitment to the NK cell lineage. **(B)** NKP cells progress to immature NK (iNK) cells, which are defined by higher expression of IL-R1. Expression of NKG2D, CD335, CD337, and CD161 are initiated and may be expressed at low levels near the end of this life-stage. **(C)** Mature NK (mNK) cells are defined by the expression of CD56 and maximal expression of NKG2D, CD335, CD337, and CD161. There are multiple subtypes of mature NK cells, which have unique functional properties, that are defined primarily via the level of expression of CD56 (i.e. CD56^bright^ *vs* CD56^dim^), and CD16 and KIR positivity. **(D)** Finally, memory-like (ML) NK cells have also been described and are defined by high levels of expression of the high affinity IL-2αβγ receptor.

NK cells are typically phenotypically defined by the expression of CD56 and lack of expression of CD3 (22). Within CD56 expressing cells, two subsets exist, defined based on level of CD56 expression: CD56^dim^ and CD56^bright^ (22). Development occurs in the bone marrow with IL-3, IL-7, c-kit ligand, and flt3 ligand coordinating to signal to CD34^+^ hematopoietic progenitor cells to commit to the NK cell lineage (**Figure 1**) (26). Then, IL-15 is needed for differentiation to CD56^+^ NK cells, which then further differentiate to CD56^bright^CD16^-^KIR^-^ then to CD56^dim^CD16^+^KIR^-^ then to CD56^dim^CD16^+^KIR^+^ subsets (**Figure 1**) (26). As compared to CD56^bright^ NK cells, CD56^dim^ NK cells are more cytotoxic likely in part due to higher expression of aKIRs and Ig-like transcripts (ILTs), which enable enhanced cytotoxic properties (22). However, the CD56^bright^ NK cell is thought to be a more potent producer of cytokines, compared to the CD56^dim^ NK cell (32). Differences in the level of expression of CD16, i.e. CD16^bright^ versus CD16^dim^, have also been shown to have important functional consequences. CD56^dim^CD16^bright^ are thought to be more cytotoxic, as CD16 mediates antibody-dependent cell-mediated cytotoxicity (22). The CD56^dim^CD16^dim^ NK cell subset degranulates at a higher level than the CD56^dim^CD16^bright^ cell, however, it is difficult to characterize where this population fits in the maturation spectrum of NK cells (33). Importantly, there is no murine homolog of CD56 expressed on mouse NK cells (22), but differential expression of the Ly49 family or CD27^high^ versus CD27^low^ expression has been used to functionally define murine NK cells (31, 34).

In the case of HCT and GVL, donor derived NK cells may identify leukemic cells that have downregulated or lack cognate HLA class I molecules to donor iKIRs (35–38) or those with surface markers that interact with NK cell activation receptors (18). After activation, NK cells conduct cell-killing through either release of cytotoxic granules loaded with perforin and granzymes, leading to target cell lysis, or through induction of Fas-L- or TRAIL- mediated target cell lysis (27, 38). NK cells also secrete pro-inflammatory cytokines (IFN-γ, TNF-α, IL-6), which contribute to both innate and adaptive immunity in the GVL reaction (38).

The kinetics of NK cell recovery after HCT highlight the importance of NK cells in early GVL reactions. NK cells are the first non-neutrophil white blood cell to recover after HCT, doing so during the period of severe lymphopenia following conditioning (39–41). This has been shown to be true with both non-PTCy and PTCy-based GVHD prophylaxis regimens (39–42). Two studies from the National Institutes of Health examined immunological recovery post-HCT in patients receiving TCD HCT from HLA-identical siblings (40, 41). In these studies, early NK recovery was noted to be the main predictor for molecular remission in patients with CML (41) and also was found to be associated with decreased relapse in patients with acute myeloid leukemia (AML), but not in patients with acute lymphocytic leukemia (ALL) (40). Importantly, early recovering NK cells after PTCy-based HCT may display unique phenotypic properties, as compared to those that recover after non-PTCy based GVHD HCT (39, 42, 43). Roberto et al. identified a population of CD56^dim^CD16^neg^ NK cells that are detected in high frequency early post-HCT with PTCy and display defective cytotoxicity secondary to increased anergy associated with increased surface levels of CD94/NKG2A, an iKIR (43). Russo et al. and Rambaldi et al. both demonstrated that early NK cells recovering after PTCy had an immature phenotype and demonstrated impaired *in-vitro* antileukemic activity (39, 42). These studies allude to the potential utility of adoptive NK cell therapy in PTCy platforms to compensate for post-HCT NK cell immaturity.

One of the earliest studies demonstrating the importance of NK cells in GVL was published by Ruggeri et al. in 2002 (37). In this analysis of 92 patients, the Perugia group compared the outcomes after TCD haplo HCT for recipients with and without KIR ligand incompatibility (37). The findings were striking. In HCT from NK cell alloreactive donors, there were no cases of graft rejection or grades II-IV GVHD, compared to 15.5% and 13.7% incidence respectively in patients who received HCT from NK cell non-alloreactive donors (37). Moreover, no patients with AML had relapsed at 5 years in the NK cell alloreactive donor group (37). The group then supplemented their clinical data with *in vivo* murine mechanistic studies. NOD/SCID mice with AML were transfused with either human alloreactive, non-alloreactive, or no NK cells (37). Only mice that received alloreactive NK cells survived, while mice that received either non-alloreactive or no NK cells died over a period of about three weeks (37). Interestingly, when mice were conditioned with TBI and subsequently infused with escalating doses of alloreactive T cells, concomitant infusion of alloreactive NK cells protected against the fatal GVHD that was seen with alloreactive T cell infusions alone (37). However, in chimeric mice, in whom recipient antigen presenting cells (APCs) were engineered to be resistant to the donor alloreactive NK cells, the protective benefit of alloreactive NK cell infusion was eliminated and the mice succumbed to fatal GVHD (37). The authors postulated that alloreactive NK cells are a primary mediator of GVL and could, in the future, be used as a form of cell therapy for host immune suppression, GVHD prevention (potential mechanisms for which are discussed below), and leukemia ablation (37).

## NK cell role in reducing non-relapse mortality after allogeneic HCT

Aside from their role in the GVL effect, NK cells have also been shown to be important in many additional post-HCT outcomes including protection against infection. After HCT, the recipient experiences many vulnerable months of profound immunosuppression (44). This period of immunosuppression is characterized by a high risk of bacterial and viral infections, including reactivation of opportunistic viruses like cytomegalovirus (CMV) (44). NK cells have an established role in anti-viral immunity, as demonstrated by the overwhelming viral infections associated with disorders marked by a congenital absence of NK cells (45). NK cells, as part of the innate immune system, play an important role in CMV control (44, 46). For example, in a murine model, induction and activation of NK cells via macrophage colony-stimulating factor (M-CSF) administration post-HCT led to protection against lethal CMV (44). Recipients of haplo HCT who experienced CMV reactivation have been noted to have higher proportions of mature CD56^dim^CD16^+^KIR^+^ NK cells (42) and faster acquisition of the activating NKG2C receptor (39), when compared to those who do not experience CMV reactivation. In fact, patients with a higher ratio of CD16^-^ NK cells to CD16^+^ NK cells at one- and two-months post HCT developed CMV reactivation at higher rates, suggesting that delayed NK cell maturation may make patients more susceptible to CMV reactivation (42). Certain KIR haplotypes have also been shown to be associated with better post-HCT control of CMV (46).

NK cells may also play an important role in the suppression and prevention of GVHD through lysis of responsible alloreactive T cells (45–48). Low total numbers of NK cells have been linked to poor GVHD outcomes including worse OS (45, 49). However, there is also data that NK cell-mediated production of cytokines may contribute to the inflammatory cascade responsible for GVHD (45). Interestingly, subtypes of NK cells have been identified in murine intestines that may play a role in protection against bacterial infections associated with development of gastrointestinal GVHD, through both cytokine-induced defenses and regeneration of the gut epithelium (47).

NK cell-mediated prevention of GVHD and infection translates to reduced NRM in patients with better NK cell recovery (50). The relationship of NK cells with protection from NRM is also suggested by the beneficial effects of HLA-B leader matching, which has been associated with less NRM and improved OS after MUD and haplo HCT (51, 52). HLA-B leader refers to the peptide encoded by exon 1 of the 7 exons that comprise the HLA-B gene (53, 54). The leader sequence of HLA class-I molecules is presented by the non-classical class I molecule, HLA-E, and stabilizes HLA-E expression on the cell surface, enhancing binding to receptors on NK cells (53, 54). It is hypothesized that HLA-B presentation by HLA-E and subsequent recognition by NK cells may contribute to improvements in NRM that have been demonstrated in several HCT settings and has opened another avenue of research to understand NK alloreactivity through HLA-matching (53, 54).

## Optimization of NK cell match and genotype

Fortunately, given safe HLA-mismatched HCT facilitated by PTCy, we now exist in an age of HCT where nearly every patient has a donor. In fact, many patients have multiple donors from whom to choose. These may include matched sibling donors (MSDs), matched unrelated donor (MUDs), MMUDs, HLA-haploidentical donors, or umbilical cord blood (UCB) donors. In this age of donor choice, there is increasing focus on refinement of selection algorithms to improve outcomes. This consists of optimization of HLA-matching, donor age, familial relationship, presence of donor specific antibodies (DSAs), CMV serostatus, and ABO match – among other factors. Much research has been conducted on how each of the aforementioned donor characteristics may differentially influence HCT outcomes. The creation of complex guidelines and algorithms that physicians may use for donor selection is still underway and how to prioritize these factors is still uncertain (52).

However, even less is known about optimization of NK cell alloreactivity between donor and recipient to facilitate amplified GVL and subsequently decrease risk of post-HCT relapse. What is known has been primarily studied in non-PTCy platforms, however, more data in PTCy-based HCT is emerging. Importantly, NK cell alloreactivity is probably more readily harnessed in the HLA-mismatched settings (55). However, the current body of literature (**Table 2**) regarding donor selection and NK cell alloreactivity is complex and in flux and there are indications that NK cells, regardless of alloreactivity, are critical in relapse prevention in all PTCy platforms (50). Despite the current discordant data, which will be examined below and is summarized in **Table 2**, the European Group for Blood and Marrow Transplantation (EBMT) includes NK cell alloreactivity as a criterion of interest in the 2019 Consensus Recommendations for Haploidentical Donor Selection (68).

**Table 2:** Summary of studies examining effects of NK cell alloreactivity on transplant outcomes.

Publication	N	Disease	HCT Dates	Data Source	Conditioning Platform	Graft Source	Donor Source	GVHD ppx	Major Findings
Ruggeri (2002) (37)	92	AML (57) ALL (35)	–	Single-center	MAC w/ATG (92)	PBSC (92)	Haplo (36)	TCD graft (92)	- NK alloreactive donors are associated with lower incidence of GF, grades II-IV aGVHD, and relapse
Leung (2004) (56)	36	Myeloid (17) Lymphoid (19)	–	Multi-center (2)	MAC w/ATG (36)	PBSC (36)	Haplo (36)	TCD graft (36)	- Receptor-ligand model better predicts risk of relapse as compared to the ligand-ligand model
Venstrom (2012) (57)	1277	AML (1277)	1989-2008	CIBMTR	MAC (1069) Non-MAC/RIC (189)	BM (107) PBSC (93)	MUD (664) MMUD (613)	CsA+MTX (346) Tac (428) TCD (349)	- KIR2DS1^+^ donors were associated with lower rates of relapse - Only KIR2DS1^+^ donors with HLA-C1 were associated with reduction in relapse - Only recipients with HLA-C1/C1 or C1/C2 benefit from KIR2DS1^+^ donors
Boudreau (2017) (58)	1328	AML (1328)	1989-2008	Single-center	MAC (1123) Non-MAC/RIC (186) Unknown (19)	BM (722) PBSC (606)	MUD (716) MMUD (612)	CsA-based (527) Tac-based (548) TCD (112) Other (141)	- KIR3DL1 and HLA-B subtype combinations (strong-, weak-, or non- inhibiting) predict risk of relapse with weak or noninhibiting pairs associated with superior incidence of relapse and OM - Donors with combined weak or noninhibiting subtypes of KIR3DL1/HLA-B and KIR2DS1/HLA-C1 had the lowest relapse rates
Schetelig (2020) (59)	2222	AML (1773) MDS (449)	2005-2017	DRST	MAC (436) Non-MAC (83) RIC (1694) *ATG or alemtuzumab (1775)	BM (98) PBSC (2124)	MUD (1727) MMUD (495)	TCD graft (15)	- KIR2DS1^+^ donors were not significantly associated with improved outcomes, including relapse, even when C1/C2 combinations were considered - No KIR3DL1/HLA-B subtype combination was associated with improved outcomes, including relapse - KIR3DL1 presence/absence had no significant effect on outcomes, including relapse
Cooley (2009) (60)	448	AML (448)	1988-2003	CIBMTR	MAC (448)	BM (397) PBSC (51)	MUD (209) MMUD (139)	–	- KIR B/x donors were associated with improved OS and RR of RFS compared to KIR A/A donors - KIR B/x donors were associated with higher incidence of cGVHD, but not aGVHD
Cooley (2010) (61)	1409	AML (1086) ALL (323)	1988-2006	CIBMTR	MAC (1409)	BM (42) PBSC (467)	MUD (687) MMUD (722)	–	- KIR B/x donor genotype is not associated with relapse protection for ALL patients - Total donor centromeric gene content was the best predictor of success with Cen-B/homozygosity being associated with decrease In relapse and improved OS
Cooley (2014) (62)	1532	AML (1532)	1988-2009	CIBMTR	MAC (1532)	BM (810) PBSC (722)	MUD (856) MMUD (676)	–	- Relapse protection with donor KIR B/x donors is improved in recipients with HLA-C1/x allotypes - An HLA-C mismatch provides additive relapse protection in transplants with KIR B/x donors and HLA-C1/x recipients
Zhao (2014) (63)	97	CML (97)	2003-2009	Single-center	MAC w/ATG (97)	BM+PBSC (97)	Haplo (97)	CsA+MTX+MMF (97)	- Recipients lacking a class I ligand for donor iKIR had increased rates of relapse and worse OS - Recipients with HLA-C1/C2 or -C2/C2 with KIR2DS1^+^ donors had reduced risk of relapse - Recipients with HLA-Bw4 with KIR3DS1^+^ donors have reduced relapse rates
Zhao (2015) (64) Cohort 1: Cohort 2:	29 188	AML (12) ALL (11) MDS (4) CML (2) AML (121) ALL (51) MDS (14)	–	Single-center	MAC w/ATG (217)	BM+PBSC (217)	Mis-matched (29) Matched (1) Mis-matched (187)	CsA+MTX+MMF (217)	- NK cell number and functional recovery is correlated with number of recipient KIR ligands - Recipients that expressed all ligands for donor iKIR had the most functional NK cells and lowest relapse rates
Zhao (2019) (65) Cohort 1: Cohort 2:	114 276	AML (58) ALL (34) CML (5) AML (203) MDS (73)	2016-2017 2012-2016	Single-center	MAC w/ATG (390)	BM+PBSC (390)	Haplo (390)	CsA+MTX+MMF (390)	- NK cell number and responsiveness is higher in pairs where donors and recipients both expressed ligands for donor iKIRs - The effect on NK cell responsiveness of expression of the 3 major KIR ligands in donor/recipient pairs is additive - Donor/recipient pairs expressing all ligands for donor iKIRs have lower relapse rates
Symons (2010) (66)	86	AML (25) ALL (7) MDS (8) CML/CMML (11) HL (7) NHL (14) MM (6)	1999-2007	Single-center	Non-MAC (86)	BM (86)	Haplo (86)	PTCy w/MMF+Tac (86)	- iKIR gene-mismatched donors were associated with improved OS, EFS, and incidence of relapse - Recipients with KIR-A/A haplotypes with donors of KIR-B/x had significantly improved OS, EFS, and NRM
Sahin (2018) (67)	96	AML (62) CML (34)	1994-2014	Single-center	MAC (79) RIC (17)	BM (20) PBSC (79)	MRD (96)	CsA+MTX (79) CsA+MMF (17)	- Matching of aKIR results in decreases in relapse rates and increases in DFS - iKIR-matching had no significant effects of relapse rates, OS, or DFS

AML, acute myeloid leukemia; ALL, acute lymphoblastic leukemia; CML, chronic myeloid leukemia; MDS, myelodysplastic syndrome; CMML, chronic myelomonocytic leukemia; HL, Hodgkin lymphoma; NHL, non-hodgkin lymphoma; MM, multiple myeloma; CIBMTR, Center for International Bone Marrow Transplant Research; DRST, Deutches Register Für Stammzelltransplantation; MAC, myeloablative conditioning; RIC, reduced intensity conditioning; ATG, anti-thymocyte globulin; PBSC, peripheral blood stem cell; BM, bone marrow; Haplo, haploidentical; MUD, matched unrelated donor; MMUD, mismatch unrelated donor; MRD, matched related donor; TCD, T-cell deplete; CsA, cyclosporine; MTX, methotrexate; Tac, tacrolimus; MMF, mycophenolate mofetil; KIR, killer immunoglobulin-like receptor; iKIR, inhibiting killer immunoglobulin-like receptor; aKIR, activating killer immunoglobulin-like receptor; HLA, human-leukocyte antigen; NK, natural killer; GF, graft failure; GVHD, graft-versus-host disease; aGVHD, acute graft-versus-host disease; cGVHD, chronic graft-versus-host disease; OM, overall mortality; OS, overall survival; EFS, event-free-survival; NRM, non-relapse survival; DFS, disease-free surivival; Cen, centromeric; ppx, prophylaxis

* Grafts were T-cell replete aside from those denoted to be TCD in the GVHD ppx section

In the case of donor optimization in HCT, KIRs are of specific interest because they are polymorphic, whereas CD94/NKG2 are not (35). Additionally, given that major histocompatibility complex (MHC) and KIR are located on chromosomes 6 and 19, respectively, they segregate and are thus inherited independently, which provides additional opportunity for donor optimization aside from HLA (35). For example, among unrelated donors and recipients who are HLA-identical it is rare to be KIR identical and only 25% of HLA-identical siblings will also be KIR identical (69).

KIR proteins are classified by their extracellular domain and the length of their cytoplasmic tail, which determines their ligand specificity and functionality, respectively (35). The nomenclature of KIRs follow this convention: KIR2Dxx and KIR3Dxx have two and three extracellular domains respectively, and KIRxDSx and KIRxDLx having short activating or long inhibitory cytoplasmic tails, respectively (35). MHC molecules act as interacting ligands for KIRs and each KIR has a cognate MHC molecule (35). These MHC molecules include HLA-A, HLA-Bw4, HLA-C, and HLA-E (29). Typing of KIR and MHC prior to HCT would provide information regarding which donor/recipient pairs possess KIR/MHC combinations that lead to alloreactivity in an effort to harness the NK GVL effect. However, KIR typing is not routinely performed at most centers or unrelated donor registries, limiting widespread study of its influences and utilization of any resulting data in clinical practice.

**Figure 2:**
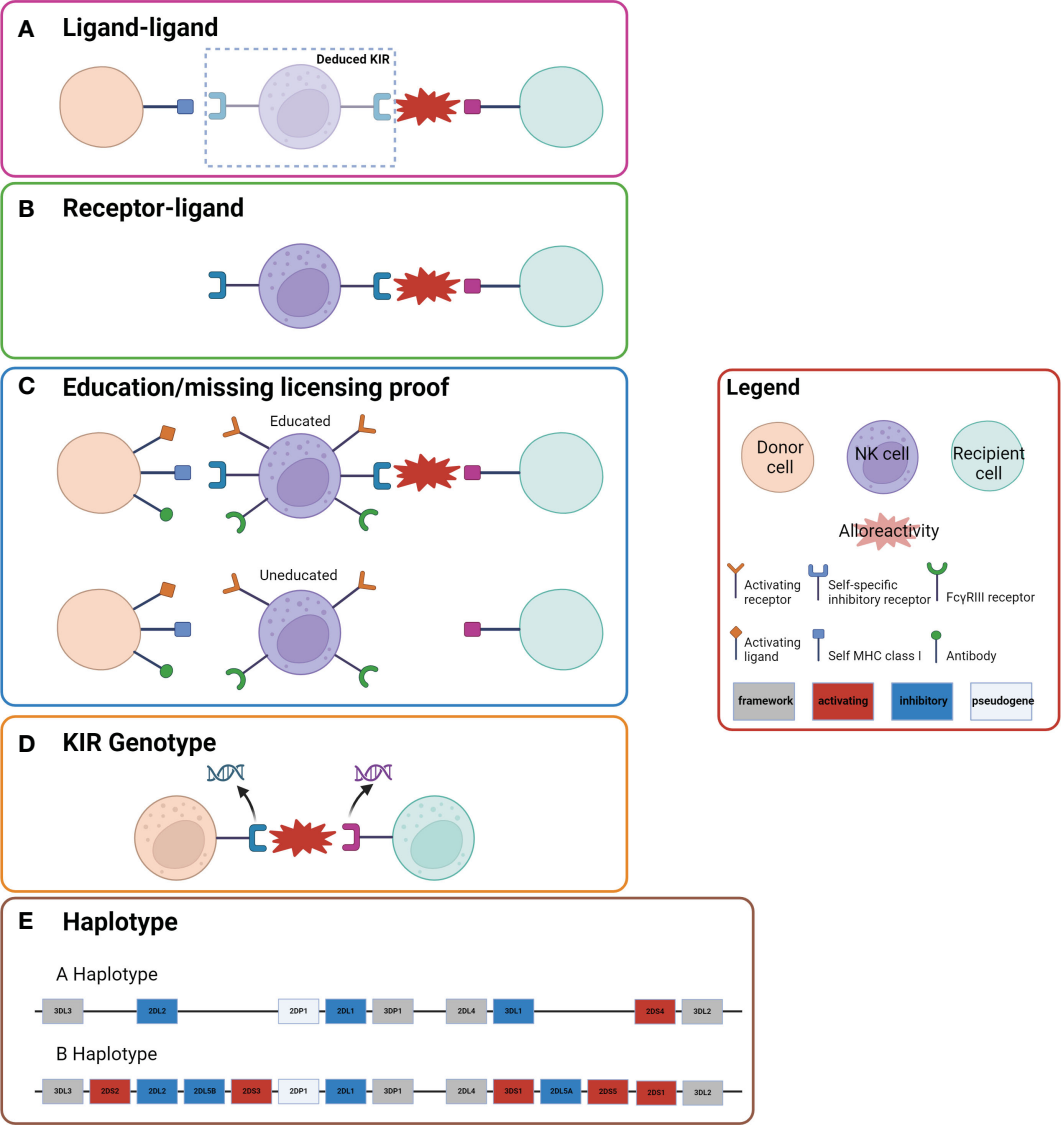
Five Models of NK Cell Alloreactivity. Representation of the five models of NK cell alloreactivity. Figure is adapted from Dhuyser et al. (36) and Boudrea et al. (70). **(A)** In the ligand-ligand model NK cell alloreactivity occurs when the recipient lacks a ligand that is present in the donor. **(B)** In the receptor-ligand model (missing-ligand model), if the KIR of the donor does not recognize the MHC molecule of the recipient there will be alloreactivity. **(C)** In the education/missing licensing proof model the process of NK cell licensing is considered. When a NK cell that has been “licensed” or “educated” based on a self-specific inhibitory receptor to a specific self MHC class I ligand encounters a recipient cell that does not have the same self MHC class I ligand, there is alloreactivity. **(D)** In the KIR genotype model certain mismatches of KIR genotypes result in alloreactivity. **(E)** The haplotype model examines the KIR haplotype of donor and recipient. Donors with KIR B haplotypes have been shown to have improved outcomes, which has been postulated to be due to more activating KIR genes.

Several models have been proposed to predict KIR compatibility between recipient and donor post-HCT (**Figure 2**). Each model places emphasis on a different component of NK cell immunobiology and the interaction between donor NK cells and recipient cells: i.e. whether the onus of the mismatch lies in interface of the receptors or the ligands. These include: the ligand-ligand mismatch model (29, 35–38), receptor-ligand/missing-ligand model (27, 35, 37), receptor-receptor mismatch/haplotype model (60), educational/missing licensing proof model (71), and NK cell genotyping (66, 67). Ultimately, the multitude of models that exist in the literature to predict NK cell alloreactivity in HCT illustrates the complexity of NK cell alloreactivity and the need for unifying research to increase its applicability.

Understanding NK cell licensing is critical to comprehending the complexities of each NK alloreactivity model. Just as T-cells undergo positive selection to prevent alloreactivity, NK cells also undergo a process so they may discriminate self from non-self called “licensing”. In a healthy person an NK cell will encounter the matching self-HLA-class I ligand for that NK cell’s specific inhibitory KIR and become “licensed” to refrain from attack on healthy self-antigens (72, 73). When licensed NK cells encounter a cell that does not express or is missing the appropriate KIR-ligand they are able to mount a response if there is also expression of ligands that interact with the aKIRs of the NK cell (72, 73). Unlicensed NK cells are not functionally competent and are hyporesponsive when compared to licensed NK cells (72, 73).

The ***ligand-ligand mismatch model*** was established in 2002 by Ruggeri et al. (29, 35–38). This model is derived primarily from the concept of “missing-self” or the activation of donor NK cells due to failure of donor iKIRs to bind to their cognate ligand on recipient target cells (29) (**Figure 2A**). This model postulates that donor NK cells will be alloreactive toward host cells when the recipient is lacking HLA class I ligands that are present in the donor (29, 35). Therefore, after HCT, donor-derived NK cells will encounter host cells that do not provide the requisite inhibitory signals, and absence of inhibitory signals leads to activation of NK cytotoxicity and a cascade of alloreactivity. As the name indicates, a comparison of the donor and recipient KIR ligands is conducted to assess alloreactivity in this model. As discussed in the proceeding section, reductions in relapse and absence of GVHD were seen in AML patients who underwent HCT from ligand-ligand mismatch donors (37).

The ***receptor-ligand mismatch/missing-ligand model*** was proposed by Leung et al. in 2004 (29, 35, 56) (**Figure 2B**). Defined by donor and recipient KIR ligand incompatibility, this model hypothesizes that if at least one KIR expressed in the donor does not recognize any of the HLA molecules of the recipient, then there will be a reduction in NK cell inhibition and therefore increased cytotoxic activity (35). An ideal donor would have at least one inhibitory KIR for which the recipient lacks a ligand, but also at least one cognate recipient ligand so that NK cells maintain some immunoregulatory function (35).

Prediction of NK cell alloreactivity based on donor KIR2DS1, KIR3DL1, and their cognate ligands has been proposed under the receptor-ligand mismatch model (57–59). KIR2DS1 is an activating KIR that has a role both in NK cell activation and tolerance via interaction with the HLA-C locus (57). More specifically, the interaction between KIR2DS1 and HLA-C1 versus HLA-C2 molecules drives NK responsiveness (57, 74–76). KIR2DS1-positive NK cells that have been exposed to HLA-C1 ligands secrete IFN-γ and are cytotoxic to target cells, especially those that express HLA-C2 (57). In a HLA-C2 homozygous host, KIR2DS1-positive NK cells are hyporesponsive– (i.e. the activating receptor KIR2DS1 causes limited NK cell function in the setting of exposure to self-ligand) (57).

In 2012, Venstrom et al. demonstrated that patients with AML who received allografts from donors who were positive for KIR2DS1 did indeed have a lower rate of relapse than those with allografts from donors who were negative for this KIR genotype (57). Additionally, recipients with an HLA-C2/C2 genotype who received allografts from KIR2DS1-postive donors did not benefit from relapse protection, while recipients with HLA-C1/C1 or C1/C2 genotypes had significantly reduced incidence of relapse (57). Similar findings were reported when KIR3DL1 and its cognate ligand HLA-Bw4 were examined (57, 58). Conversely, a more recent study in a more varied population had negative results when examining KIR2DS1, KIR3DL1, and their cognate ligands as predictors of NK alloreactivity (59). Importantly, patients in this more modern study underwent a variety of conditioning regimens intensities, peripheral blood stem cell (PBSC) grafts dominated, and anti-thymocyte globulin (ATG) was used as GVHD prophylaxis in the majority of patients (59). In contrast, the previously mentioned prior studies examined patients who in the majority received MAC, no ATG, and bone marrow (BM), less than 1/3 of which were TCD, grafts (57, 58).

Another model more recently proposed by Nowak et al. in 2014 is the ***education/missing licensing proof model*** (71) (**Figure 2C**). This model considers both donor and recipient HLA class I molecules, the KIR type of donor, and the education process required for NK cells to become competent or “licensed.” Specifically, for a NK cell to mature and possess the ability to become alloreactive, it must first become “licensed” via its iKIR interacting with its cognate ligand (35). In the case of HCT, alloreactivity is achieved when a licensed donor NK cell interacts with the recipient cells that lack its cognate ligand, therefore allowing for NK cytotoxicity and GVL effects (35).

Attempts in murine models have been made to understand whether donor or recipient MHC may play a more dominant role in NK cell alloreactivity or education (77–80). For instance, educated NK cells from wild-type mice transferred into MHC-deficient mice have been shown to be significantly less responsive in multiple studies (78, 79). On the other hand, when educated NK cells were depleted based on prediction from donor MHC haplotypes and transfused into a wild-type MHC-mismatched HCT murine model there was increased susceptibility to CMV infection (80). Human studies to tease out the respective effects of donor and recipient NK-cell education and alloreactivity have been mixed (63, 64, 81), with some indicating that donor HLA genotype may shape education (81) and others leaning towards host (63, 64) as driving NK cell alloreactivity. For instance, Zhao et al. showed that donor/recipient pairs that expressed all ligands for donor inhibitory KIRs had the lowest relapse rate after haplo-HCT, which they attributed to better education and function of NK cells (65). All in all, it is likely that both donor and recipient NK cells cooperate to maintain and adjust NK cell responsiveness and augment GVL effects.

***KIR genotyping*** involves understanding whether a specific donor and/or recipient KIR gene, or mismatch between the two, may be associated with improved outcomes and reduced post-HCT relapse (**Figure 2D**). Data regarding the effects of KIR genotype on HCT outcomes is discrepant. Differences in iKIR gene content in haplo HCT were associated with improved OS, EFS, and lower relapse rates compared to pairs with identical iKIR gene content in both lymphoid and myeloid diseases in a study by the Johns Hopkins group (66). In contrast, another study found the inverse: that KIR matching in MSD HCT was associated with less relapse (67). As in many things, these results may be different due to the different GVHD prophylaxis strategies employed with PTCy in the former study and cyclosporine with methotrexate or mycophenolate mofetil in the latter. Thus, further analyses are required to fully elucidate the impact of KIR on HCT outcomes, as these effects are likely platform specific.

The last model we will discuss, the ***receptor-receptor mismatch/haplotype based model***, was proposed by Gagne et al. in 2002 (82) (**Figure 2E**). This model is defined by donor KIR and recipient KIR incompatibility. Since KIR mismatch is closely related to the presence of B-haplotype activating genes, this model may alternatively be defined as a mismatch between donor and recipient for KIR B haplotypes (29). Briefly, KIR genes are organized and inherited via group A and group B haplotypes. The KIR B haplotype contains variable polymorphic gene content including one or more activating genes, while the KIR A haplotype contains preserved gene content, which does not include activating genes (60). Both haplotypes have distinctive centromeric (Cen) and telomeric (Tel) gene-content motifs that may be used in further classification (61).

Cooley et al. examined how donor KIR genotype would affect outcomes after TCR unrelated donor (URD) HCT in multiple consecutive studies (60–62). First, in 2009, they found a significantly improved 3-year relapse-free survival (RFS) when using donors who had at least one KIR B haplotype-defining loci (i.e. B/x) (60). These results were also replicated in later studies by the same (61) and other groups (83–85). Importantly, these studies include a variety of platforms: TCD grafts (83), PBSC grafts (84, 85), and varied conditioning regimens, including nonmyeloablative (NMA) (66) and RIC (84, 85). Interestingly, having multiple KIR B haplotype-defining loci provided no additional benefit beyond one KIR B-haplotype (60). When individual donor KIR genes were examined, KIR2DL2 (p=.07) and KIR2DS2 (p=.02) were shown to be independent predictors of OS (60). They also found that total centromeric and telomeric gene content were predictors of relapse and OS (61). Cen-B/homozygosity had the strongest independent effect and was associated with decreased relapse and improved OS (61). Expanding on their 2009 and 2010 findings, Cooley et al. examined the effect of the inhibitory C1 and C2 receptors, which are encoded by genes in the centromeric region, on the protection provided by donor KIR-B haplotypes in HCT (62). Relapse protection associated with donor KIR B was enhanced in recipients with one or more C1 bearing HLA-C allotypes (62). This suggests that the interaction between donor KIR B and recipient C1 may be a mechanistic explanation for protection against relapse.

A recently proposed model by a London group that has studied NK genotyping and its influence on chronic infection, suggested that the donor iKIR-ligand genotype measured by the count of iKIR-ligand gene pairs will impact relapse and GVHD in HCT (86). They posited that higher numbers of iKIR-ligand gene pairs will prolong memory CD8^+^ T cell survival and that this would prevent relapse, albeit possibly with an increase in risk of GVHD (86). This model puts the onus back on the T cells as the primary mediators of GVL and hypothesizes that the discrepant findings in prior studies that examined the effects of NK alloreactivity may be due to failure to account for this T cell interaction (86). While intriguing, this model has yet to be examined in HCT and we will await the forthcoming studies to review it further.

## NK cell alloreactivity in PTCy

Most of the studies examining the prior NK alloreactivity models include a significant portion of patients who received ATG or TCD strategies – and therefore many of the aforementioned models and studies may not be as relevant in the modern age of HCT that is marked by increasing use of PTCy. Nevertheless, NK cell alloreactivity has been demonstrated to be associated with outcomes in several studies of HCT with PTCy (54, 66, 87, 88). While Shimoni et al. showed that KIR ligand mismatching was significantly associated with worse OS and a trend for higher incidence of relapse (89), the Georgia group showed that HCT with donor/recipient pairs who expressed at least one KIR receptor-ligand mismatch were associated with improved OS and reduced risk of relapse (87). Similarly, both the Georgia group and Symons et al. also showed that HCT from donor-recipient pairs with mismatched KIR haplotypes were associated with improved OS, EFS, and decreased relapse rates (66, 87). The Georgia group also showed that recipients with M+ genotype (-21M HLA-B) at the B-leader sequence had decreased relapse rates. They speculated that this was due to B-leader mediated expression of HLA-E that then recognizes NKG2A, thereby contributing to education of NK cells (54).

Symons et al. also examined the effects of KIR haplotypes in patients who received NMA haplo HCT with PTCy and found that recipients with KIR A haplotype who received grafts from KIR B haplotype donors had improved OS (66). In addition, recipients with iKIR mismatched haplotypes had significantly improved OS, EFS, and decreased relapse rate when compared to iKIR matched HCT (66). Illustrating the platform-dependent effects, Cooley et al. did not find a benefit of iKIR mismatching in patients receiving TCR HCT, in the absence of PTCy, despite demonstrating benefits in TCD HCT (60). These distinct outcomes may be a result of differences in expression of KIRs and in cytokine production between TCD and TCR grafts; for instance, NK cells after TCR grafts express fewer KIRs and produce more IFN-γ (90). Ultimately, NK cell alloreactivity may be more important in TCD HCT where T cells play less of a role in GVL (55). As such, all models must be tested in different platforms to determine their applicability and one model will likely not fit all.

## Adopting the NK cell for enhanced GVL effects: an alternative to donor lymphocyte infusion?

While DLI, a major cellular treatment of post-HCT relapse, can be associated with remission rates of 10-15%, it also comes with a high risk of GVHD, which limits its use prophylactically (91–96). Prophylactic adoptive transfer of NK cells is an attractive alternative given its potential to increase GVL without significantly increasing the risk of GVHD. Haplo NK cell infusion was examined in the non-HCT setting where it induced remission in a few patients with poor-prognosis AML without significant toxicity (97). Moreover, adoptive transfer of NK cells has shown to be both feasible and safe in multiple HCT clinical trials, summarized in **Table 3** (30, 37, 91). For instance, one group demonstrated safety of autologous NK cell infusion after HCT, but noted that there were no significant improvements in disease outcomes, which they attributed to a lack of KIR mismatching (97, 98). While adoptive transfer of donor NK cells has shown feasibility (99–102, 111) and efficacy (99), with reductions in progression of disease (101, 102) without increasing GVHD (100, 101), enhancing the GVL effects of adoptive NK cell transfer requires further optimization. For instance, *in vitro* activation (103, 104, 111) and expansion of NK cells in culture or via *in vivo* administration of IL-2, other cytokines, or adjuvant immunotherapies are avenues under study to increase NK cell expansion and/or persistence (97, 105, 106, 111, 112).

**Table 3:** Summary of studies examining the novel use of NK cells a therapeutic strategy for anti-tumor effects.

Publication	Phase	N	Study Population	Study Description	Findings
Miller (2005) (97)	Phase I/II	43	- Non-transplant setting - Patients with metastatic melanoma, RCC, refractory Hodgkin disease after auto-HCT, or poor prognosis AML	- Comparison of lower intensity (Lo-Cy/mPred or Flu) *vs* high intensity (Hi-Cy/Flu) pre-adoptive NK cell infusion - Adoptive transfer of TCD product from haplo donors was followed by daily IL-2 injections for 14 days for *in vivo* NK cell expansion	- *in vivo* expansion of NK cells did not occur in the low immunosuppression dose groups, but did occur in the high immunosuppression dose group - 5 patients with poor-prognosis AML achieved CR - Adverse effects included: anemia, neutropenia - No evidence of aGVHD or cGVHD after cell infusions in any group
Burns (2003) (98)	Phase I/II	57	- Patients with relapsed lymphoma or metastatic breast cancer post-auto-HCT without evidence of disease progression	- All patients received low-dose s.c IL-2 prior to either arm - Arm 1: Infusion of *ex vivo* IL-2-activated autologous NK cells - Arm 2: Patients received supplemental IL-2 bolus infusions	- There was a greater than 10-fold increase in NK cells during the IL-2 treatment period - There was an increase in cytotoxicity in PBMNCs obtained D +1 of either infusion of IL-2 activated cells or iv IL-2 boluses - No improvement in disease outcomes in either group in matched-pair analysis
Passweg (2004) (99)	Phase I	5	- Patients with high-risk myeloid malignancies who received a haplo-HCT 3-12 months prior to study and had either mixed chimerism, impending graft failure, or early relapse	- Test of feasibility of NK cell 2-step *ex vivo* procedure w/purification via immunomagnetic TCD and NK cell enrichment	- Purification technique achieved NK cell purity of about 97% and TCD by 3.5 logs - 4 of 5 patients remained in remission 8-18 months after first NK-DLI, 2 patients achieved increasing donor chimerism, chimerism stabilized in one patient - One patient experienced early relapse - No immediate adverse reactions during NK-DLI infusion - No evidence of GVHD post-NK cell infusion
Yoon (2009) (100)	Phase I	14	- Patients with AML or MDS who underwent HLA-mismatched HCT	- Adoptive transfer of donor *ex vivo* (via culture w/IL-15, IL-21, and hydrocortisone) generated NK cells generated from previously collected CD34^+^ selected cells were administered (one dose) between D +43 through D +50	- *Ex vivo* generated NK cells demonstrated cytotoxicity and higher IFN-γ and TNF-α secretion compared to freshly collected NK cells - No signs of acute toxicity during NK-DLI infusion - One patient developed aGVHD and four patients developed cGVHD during the post-HCT period
Choi (2014) (101)	Phase I/II	41	- Patients with hematological malignancies that underwent haplo-HCT	- Adoptive transfer of TCD *ex vivo* generated NK cells, infused twice at 2 and 3 weeks after HCT at escalating doses	- Significant reduction in leukemia progression compared to historical patients receiving haplo-HCT with same conditioning regimen - No difference in engraftment, grades II-IV aGVHD, moderate to severe cGVHD, and TRM compared to historical patients receiving haplo-HCT with same conditioning regimen - No acute toxicities observed with NK-DLI - Nine patients developed aGVHD and ten patients developed cGVHD
Choi (2016) (102)	Phase II	51	- Patients with acute leukemia who underwent haplo-HCT	- NK-DLI administered on days +6, +9, +13, +20 of HCT	- Compared to 2014 study with NK-DLI on days +14 and +21, additional NK-DLI did not significantly improve leukemia progression rates - Adverse effects included: fever, weight gain, hyperbilirubinemia
Romee (2017) (103)	Pre-clinical/Phase I	9	- Patients with relapsed or refractory AML who were not eligible for immediate HCT	- Infusion of allogeneic haplo IL-12, IL-15, IL-18 preactivated NK cells after preconditioning with flu/Cy, with low dose IL-2 administered post infusion	- Human memory-like NK cells have enhanced IFN-γ production and cytotoxicity against leukemia cell lines *in vitro* - Human memory-like NK cells xenografted into mice substantially reduced AML burden and improved OS - Recipients infused with donor memory-like NK cells exhibited enhanced numbers of IFN-γ producing NK cells in ex vivo studies - Four patients experienced CR/CRi and one patient experienced MLFS
Song (2017) (104)	Pre-clinical	–	- Murine model of allo-HSCT	- Infusion of IL-12/18 *vs* IL-12/15/18 preactivated NK cells into a murine model of allo-HCT	- IL-12/18 and IL-12/15/18 preactivated donor NK cells had a mature phenotype - IL-12/18 and IL-12/15/18 preactivated donor NK cells mediated stronger GVL effect than control NK cells in a murine allo-HCT model - Only preactivated IL-12/18 preactivated NK cells both enhanced GVL effect and mitigated aGVHD
Rubnitz (2010) (105)	Phase I	10	- Pediatric patients who had completed chemotherapy and in first CR	- Infusion of KIR-HLA mismatched NK cells followed by six doses of IL-2 after conditioning with low dose Cy/Flu	- Patients had transient engraftment for a median of 10 days and experienced expansion of KIR-mismatched NK cells - All patients remained MRD-negative at 1, 2, and 4 months after NK infusion - All patients remained in remission with median follow-up of 964 days - No evidence of GVHD
Bachanova (2014) (84)	Phase II	42	- Patients with relapsed or primary refractory AML	- Lymphodepleting Cy/Flu was followed by NK cell infusion and IL-2 administration - 15 patients received T_reg_ depleting IL-2-diptheria fusion protein	- Patients receiving IL2DT experienced improved CR rates at days +28 and DFS at 6 months - Magnitude of T_reg_ depletion correlated with successful donor NK cell expansion - Grades 1-2 nonhematologic toxicities* were common with NK cell infusions and only one patient experienced a grade 4 hypersensitivity reaction - No infusional toxicity was associated with IL2DT - No patients developed aGVHD
Curti (2016) (106)	Phase I	17	- Patients with AML in first CR	- Infusion of NK cells from haplo-KIR-ligand-mismatched donors after flu/Cy chemotherapy, followed by IL2	- Higher number of alloreactive NK cells in infusional product was associated with prolonged DFS - With median follow up of 22.5 months (range 6-68 months) 9 of 16 patients remained disease free - 3/3 patients who were MRD+ at time of NK cell infusion achieved molecular CR of 9, 4, and 8+ months - No patients developed GVHD
Ciurea (2017) (107)	Phase I	13	- Patients with high-risk AML, MDS, or CML	- Patients received haplo-HCT and mbIL21 expanded donor NK cells were infused on days -2, +7, and +28	- No infusional reactions or dose-limiting toxicities occurred with mbIL21 expanded NK cell infusions - One patient died of NRM and one patient relapsed - Seven patients developed grades I-II aGVHD, none developed grades III-IV aGVHD or cGVHD
Shah (2015) (108)	Phase I	9	- Patients with “ultra-high-risk” solid tumors	- Adoptive transfer of donor-derived IL-15/4-1BBL-activated NK cells (aNK-DLI) were infused following HLA-matched, TCD, NMA HCT	- Five of nine transplant recipients experienced aGVHD following aNK-DLI, with grade IV aGVHD occurring in 3 patients -T-cell dose was below threshold for GVHD, implicating the aNK-DLI product
Shapiro (2022) (91)	Phase I	6	- Patients with myeloid malignancies who relapsed after haplo-HCT	- Lymphodepleting chemotherapy was followed by infusion of donor-derived CIML NK cells, followed by systemic IL-2 for 7 doses	- 4 of 6 patients responded to therapy, with three of these patients achieving CR - NK cells expanded to median peak of 10-fold (range, 10- to 50- fold) persisted for several months - Adverse effects of CIML NK infusion included: fever, grade 2 CRS (n=1), pancytopenia (n=4), transient increase in ALT/AST (n=1) - No evidence of aGVHD or cGVHD
Jaiswal (2019) (109)	Phase I	30	- Patients with relapsed/refractory leukemia	- Patients received haplo-HCT with PTCy and CTLA4IG primed DLIs were given on days -1, +7, +21, and +35	- CTLA4Ig primed DLI was associated with an upregulation mature NK cells - CTLA4IG-DLI was associated with a significant reduction in aGVHD compared a similar cohort of patients who received DLI
Liu (2020) (110)	Phase I/II	11	- Patients with relapsed or refractory CD19-postive cancers	- CAR-NK cells were created via transduction with retroviral vector expressing anti-CD19 CAR, IL-15, and inducible caspase9 and infused at escalating doses after lymphodepleting chemotherapy	- Eight patients responded to treatment with CAR-NK cells with 7/8 patients having CR - Infused CAR-NK cells persisted for at least 12 months - No evidence of CRS, neurotoxicity, or GVHD

haplo, haploidentical; HCT, hematopoietic cell transplant; CIML, cytokine-induced memory like; NK, natural killer; CRS, cytokine release syndrome; GVHD, graft-versus-host disease; aGVHD, acute graft-versus-host disease; cGVHD, chronic graft-versus-host disease; RCC, renal cell carcinoma; auto, autologous; AML, acute myeloid leukemia; Lo-Cy/mPred, low dose cyclophosphamide/methylprednisolone; Flu, fludarabine; Hi-Cy/flu, high dose cyclophosphamide/fludarabine; TCD, T-cell depleted; CR, complete remission; s.c., subcutaneous; PBMNC, peripheral blood mononuclear cells; iv, intravenous; DLI, donor lymphocyte infusion; MDS, myelodysplastic syndrome; HLA, human leukocyte antigen; TRM, transplant related mortality; OS, overall survival; allo, allogeneic; GVL, graft-versus-leukemia; Treg, T-regulatory; MRD, minimal residual disease; KIR, killer cell immunoglobulin-like receptor; IL2DT, IL-2-diptheria toxin; DFS, disease-free survival; CML, chronic myeloid leukemia; mbIL21, membrane-bound interleukin 21; NRM, non-relapse mortality; NMA, nonmyeloablative; ALT, alanine aminotransferase; AST, aspartate transaminase; PTCy, post-transplantation cyclophosphamide; CAR, chimeric antigen receptor

*fever, chills, hypertension/hypotension, dyspnea, hypoxemia, headaches

Exploration of methods to expand and/or activate NK cells have been numerous. IL-2 administration has been utilized to enhance NK cell activity and persistence in many studies (91, 97, 98, 103, 105, 106, 112), given that it augments NK cell activity (113) and is one of the second signals needed for CD56^bright^ NK subset to product IFN-γ (32). Importantly, although IL-2 expands and activates NK cells it also expands T regulatory cells (T_regs_), which have an immunosuppressive effect on NK cells (32). When T_regs_ were depleted in conjunction with NK cell infusion via IL-2 -diphtheria fusion protein, NK cell expansion was more successful, illustrating that manipulation of the immune milieu may be vital and infusion of IL-2 alone may be insufficient to improve effectiveness or persistence of adoptive NK cell therapy (84). Alternative NK cell infusion schedules, i.e. earlier and more frequent infusions to harness cytotoxic benefit, have been explored without additional benefits, but with increased infusional reactions (102). Additionally, alternative cytokines have been trialed either *in vivo* or *ex vivo* to enhance activation of NK cells, including IL-12, IL-15, and IL-18 (103, 104). In a murine model, IL-12/18 and IL-12/15/18-preactivated NK cells were shown to produce a primarily phenotypically mature NK cell and mediate stronger GVL effect as compared to non-activated NK cells (104). However, in a phase I study, NK cell pre-activation with IL-15/4-1BBL resulted in unacceptable rates of severe aGVHD (108). Alternatively, Miller et al. demonstrated that successful *in vivo* expansion of NK cells, even with subsequent IL-2 injections, may be dependent on lymphodepleting chemotherapy prior to NK cell infusion (97). Clearly, there are multiple strategies that may improve the effectiveness of adoptive NK cell therapy and further work will be needed to optimize these platforms.

NK cell adoptive transfer may be a particularly attractive strategy to prevent relapse after HCT with PTCy given the impairment in maturation and function of NK cells early after administration of Cy (39, 42, 50). A phase 1 clinical trial, conducted by Ciurea et al., explored the safety and feasibility of using NK cells expanded by stimulation with K562 feeder cells infused before or after haplo-HCT with PTCy to decrease the risk of leukemia relapse (107). NK cells examined at day +28 in were more active, as measured by secretion of TNF-α and IFN-y, and displayed a functional shift towards cytotoxicity (107). Importantly, there were no infusion reactions and no increased incidence of severe acute or chronic GVHD and a very low relapse rate when compared to historical controls (107).

Another attractive option to enhance GVL is cytokine-induced memory-like (CIML) NK cells, which can be generated via incubation with IL-12, IL-15, and IL-18 and have been shown to have enhanced cytotoxicity against target leukemia cells in preclinical studies (114). These CIML NK cells have also been shown to be capable of enhanced survival, expansion, and avoidance of anergy in murine studies (91, 103, 114). Given these encouraging pre-clinical findings, a phase I trial of CIML NK cells infused in patients with relapsed myeloid malignancies after haplo-HCT was conducted and demonstrated a reduction in disease burden in 4 out of 6 patients with sustained responses (91). No patient developed severe cytokine release syndrome (CRS), neurotoxicity, or any GVHD (91). Additionally, CIML NK cells were detected several months after infusion, perhaps making this strategy one of the most effective thus far to improve NK persistence (91). In all, these early studies of adoptive NK transfer after HCT are encouraging.

In another avenue of research, CTLA4 inhibition by abatacept has been employed to mitigate the risk of GVHD after traditional DLI and, in that setting, the efficacy of DLI may rely on NK cells (109, 115, 116). CTLA4Ig-IG binds to CD80/CD86 receptors on APCs, blocking T cell activation via CD28-B7 ligand interaction (109). In a melanoma murine model, CTLA4Ig infusion prolonged survival, but that benefit was lost when NK cells were depleted prior to CTLA4Ig infusion (116). Sequential co-infusions on days +7, +21, +35 of CTLA4-Ig and DLI in PTCy-based haplo-HCT was shown to be associated with significant surges of NK cells, a low incidence of GVHD and NRM, and failure to proliferate NK cells was associated with subsequent disease progression (115). These early results underscore that novel therapies may be able to enhance NK cell activity, while avoiding the risk of GVHD.

## CAR-NK cells

Anti-CD19 CAR-T cell therapy has been demonstrated to induce high rates of durable remissions in adult patients with lymphoma and chronic lymphocytic leukemia as well as in children with acute lymphoblastic leukemia (ALL) (117–126). However, in adults with ALL, CAR-T cell-induced remissions tend to be much less durable and require consolidative HCT to reduce subsequent relapse (127–129). Manufacturing autologous CAR-Ts for patients with acute leukemia is difficult in part due to the reduced lymphocyte counts associated with relapsed leukemia treated with multiple cycles of cytotoxic lymphodepleting chemotherapy (130). However, “off-the-shelf” NK cells, which do not require full HLA matching, may be collected from unrelated donors, and engineered to express anti-CD19 CAR, may be an avenue to circumvent the potential obstacle of a poor lymphocyte harvest when manufacturing autologous CAR-T cells (130). Just as in adoptive NK cells, co-culture with NK cell-stimulating interleukins like IL-2 or IL-15 is under study as a way to further augment CAR-NK persistence. MD Anderson conducted a phase I/II clinical trial of anti-CD19 CAR-NK cells, which also expressed IL-15 and an inducible caspase 9 safety switch in patients with relapsed CD19-positive cancers (130). They demonstrated that CAR-NK cells were not associated with development of CRS or GVHD, that they were capable of inducing CR in 7 of 11 examined patients, and that they persisted at 1-year (130). Similarly, pre-clinical data demonstrated that NK CARs expressing IL-15 and IL-15/IL-15Rα were associated with improved anti-tumor activity and persistence (97, 110). Attempts to expand CAR-NK cell platforms to other hematologic malignancies are underway (131). The absence of CRS or GVHD after infusion of CAR-NK cells makes them particularly appealing as a strategy to prevent relapse after HCT in high-risk patients.

## Concluding thoughts

Decades of progress in HCT have heralded a modern age in which decreased incidences of graft failure, GVHD, and NRM have made HCT safer for patients with hematologic malignancies (4). Additionally, modern GVHD prophylaxis, including PTCy and abatacept, has expanded the donor pool allowing virtually all patients to have access to this life-saving therapy and unlocking an age of donor choice (132). As such, prevention and treatment of post-HCT relapse remains the biggest area of unmet clinical need (4, 6, 7). The GVL reaction, and particularly the role of NK cells in this reaction, is an exciting and dynamic area of research with broad potential application.

While NK alloreactivity has been associated with decreased leukemia relapse in many studies over many decades, it has yet to be implemented broadly and KIR genotyping is not routinely performed (133). Just as we are beginning to refine our selection of the optimal donor thru a deeper understanding of HLA (52), further research regarding the immunobiology of NK cells will one day allow us to define an ideal donor based on NK cell genotyping and match. Until that day is reached, we must examine NK alloreactivity in a platform-specific manner given that novel platforms, such as PTCy, will most likely influence the effects of NK alloreactivity, just as they have changed the implications of HLA-matching (35, 36, 39, 42, 50). We anticipate that harnessing the effects of allogeneic NK cells utilizing KIR mismatched donors will become increasingly important with the growing use of PTCy (134).

Beyond donor selection, newly emerging data regarding the safety and feasibility of adoptive NK cells for prevention and treatment of post-HCT relapse may represent yet another era in designer DLI with the potential to separate GVL from GVHD (91, 97, 98, 103, 105, 106, 112). Given that NK cells may exhibit functional differences after PTCy (39, 50), NK cell adoptive therapy may be leveraged to support maturation and function of NK cells in patients either at high-risk for relapse or with inadequate NK cell recovery or functional markers post-HCT. Moreover, changes to the PTCy platform to promote recovery of NK cells such as reducing the dose of PTCy or minimizing calcineurin inhibitor use are promising therapeutic options to augment the GVL reaction of HCT (135). Finally, allogeneic CAR-NK cells have the potential to overcome the manufacturing issues associated with autologous CAR-T, while lacking the risk of GVHD associated with allogeneic CAR-T cells and increasing their applicability to HCT platforms (110, 130).

NK cells provide a promising, but so far underutilized, platform for major improvements in HCT. As we define the ideal donor based on factors including KIR alloreactivity, we may soon be able to individualize donor selection algorithms and usher in the era of personalized HCT. In time, we anticipate that planned and preemptive adoptive NK and CAR-NK therapies will be commonplace, decreasing relapse and NRM, to improve post-HCT survival for the next generation of HCT recipients.

## Author contributions

AH: Writing – review & editing, Writing – original draft, Conceptualization. SM: Writing – review & editing, Writing – original draft, Conceptualization.
